# Supplementary material for: Dissipative quadratic soliton in the cascaded nonlinearity limit
Source: Nat Commun. 2025 Dec 8;17:502. doi: 10.1038/s41467-025-67195-5 (PMC12804688; doi:10.1038/s41467-025-67195-5)
Supplement: Supplementary file 1 — Supplementary Information [file 41467_2025_67195_MOESM1_ESM.pdf]

Supplementary Information for
"Dissipative quadratic soliton in the cascaded nonlinearity limit"

Mingming Nie[1, 2*] Jonathan Musgrave[1] and Shu-Wei Huang[1,3 *]

[1]*Department of Electrical, Computer and Energy Engineering, University of Colorado Boulder, Boulder, Colorado, USA*

[2]*Key Laboratory of Optical Fiber Sensing and Communications (Education Ministry of China), University of Electronic Science and Technology of China, Chengdu, China*

[3]*3DIC Research Center, National Yang Ming Chiao Tung University, Hsinchu, Taiwan*

[*]*Corresponding author:* mingming.nie@uestc.edu.cn, shuwei.huang@colorado.edu

This Supplementary Information for "Dissipative quadratic soliton in the cascaded nonlinearity limit" provides additional information for the main text.

- In Supplementary Note 1, we present the theory of DQS in a singly resonant cavity-enhanced SHG system and provide the list of simulation parameters.
- In Supplementary Note 2, we provide the experimental details of the pulsed pump laser.
- In Supplementary Note 3, we present the numerical study of the effect of GVM on bright DQS.
- In Supplementary Note 4, we present the numerical study of the effect of phase mismatch on bright DQS.
- In Supplementary Note 5, we present the numerical study of the effect of GVD on bright DQS.
- In Supplementary Note 6, we provide the single sideband phase noise spectra of bright DQS and platicon.
- In Supplementary Note 7, we present the intensity autocorrelation traces of bright DQS and platicon.
- In Supplementary Note 8, we present the optical spectrum of the green frequency comb generated through SFG between the FF and SH.
- In Supplementary Note 9, we present the simulated DQS generation dynamics with the pulsed pump.
- In Supplementary Note 10, we present the numerical study of the effects of GVM and phase mismatch on platicon.
- In Supplementary Note 11, we present the study of the effect of pump desynchronization on bright DQS and platicon.
- In Supplementary Note 12, we present the numerical study of the effect of pump pulse shape on bright DQS and platicon.
- In Supplementary Note 13, we present the numerical study of the trapping of bright DQS by the pulsed pump.

1. Theory of DQS in a singly resonant cavity-enhanced SHG system

The field evolution in the retarded time frame in cavity-enhanced second-harmonic generation obeys the coupled equations [1–3]:

$$\frac{\partial}{\partial z}A = \left[-\frac{\alpha_{c1}}{2} - i\frac{k_1''}{2}\frac{\partial^2}{\partial\tau^2}\right]A + i\kappa B A^* e^{-i\Delta k z} \quad (1)$$

$$\frac{\partial}{\partial z}B = \left[-\frac{\alpha_{c2}}{2} - \Delta k'\frac{\partial}{\partial\tau} - i\frac{k_2''}{2}\frac{\partial^2}{\partial\tau^2}\right]B + i\kappa A^2 e^{i\Delta k z} \quad (2)$$

and the boundary conditions:

$$A_{m+1}(0,\tau) = \sqrt{1-\theta_1}A_m(L,\tau)e^{-i\delta_1} + \sqrt{\theta_1}A_{in} \quad (3)$$

$$B_{m+1}(0,\tau) = 0 \quad (4)$$

where A is the FF envelope, B is the SH field envelope, A_{in} is the CW driven field, $\alpha_{c1,2}$ are the propagation losses, Δk is the wave-vector mismatch, $\Delta k'$ is the GVM between the SH field and FF field, and $k''_{1,2}$ are the group-velocity dispersions (GVDs), L is the nonlinear cavity length, θ_1 are the coupler transmission coefficients and δ_1 is the FF resonance phase detuning. $\kappa = \sqrt{2}\omega_0 d_{eff}/\sqrt{A_{eff}c^3n_1^2n_2\varepsilon_0}$ is the normalized second-order nonlinear coupling coefficient, where ω_0 is the FF center frequency, d_{eff} is the effective second-order nonlinear coefficient, A_{eff} is the effective mode area, c is the speed of light, ε_0 is the vacuum permittivity, and $n_{1,2}$ are the linear refractive indices. Higher-order dispersion and nonlinearity are both neglected for simplicity.

Under the mean field, low pump propagation loss, and good cavity approximations, Eqs. (1)-(4) can be simplified into a single mean-field equation for the fundamental field:

$$t_R\frac{\partial}{\partial t}A = \left(-\alpha_1 - i\delta_1 - i\frac{k_1''L}{2}\frac{\partial^2}{\partial\tau^2}\right)A - (\kappa L)^2A^*[A^2 \otimes I(\tau)] + \sqrt{\theta_1}A_{in} \quad (5)$$

where t is the "slow time" that describes the envelope evolution over successive round-trips, t_R is the roundtrip time, τ is the "fast time" that depicts the temporal profiles in the retarded time frame, and $\alpha_1 = (\alpha_{c1}L + \theta_1)/2$ is the total linear cavity loss of the fundamental field. The fourth term on the right-hand side is the effective third-order nonlinearity $I(\tau) = \mathcal{F}^{-1}[\hat{I}(\Omega)]$ where the nonlinear response function [1–3]

$$\hat{I}(\Omega) = \frac{1-e^{-ix}-ix}{x^2} \quad (6)$$

describes the dispersion of the effective third-order nonlinearity. Here, $x(\Omega) = \xi - D_1\Omega - D_2\Omega^2$ where Ω is the angular frequency with respect to the FF, $\xi = \Delta kL$ is the phase mismatch, $D_1 = \Delta k'L$ is the temporal walk-off between the SH field and FF, $D_2 = k_2''L/2$ is the group delay dispersion (GDD) of SH field. To shed light on the nonlinear response function, we separate $\hat{I}(\Omega) = P(\Omega) - iQ(\Omega)$ into the real and imaginary parts to individually examine their effects. Here, $P(\Omega)$ and $Q(\Omega)$ resemble the dispersive effective two-photon absorption (ETPA) and the dispersive effective Kerr nonlinearity (EKN), respectively.

When the temporal walk-off D_1 is considered, the frequency-dependent nonlinear response function $\hat{I}(\Omega)$ is evidently perturbed and asymmetry occurs in both $P(\Omega)$ and $Q(\Omega)$. As shown in Supplementary Figs. 1 and 2, we plot the dispersive effective third-order nonlinearity as a function of the GVM with ξ=2π and ξ=-2π, respectively. The dashed lines in Supplementary Figs. 1 and 2 indicate the 3-dB soliton bandwidth of 1.5 THz, corresponding to a 200-fs sech^2 pulse. With ξ=2π, k''_2=372 fs/mm^2 and L=25 mm, two resonant ETPA peaks and associated nonlinear phase anomalies can be found from the two real roots of $\xi - D_1\Omega - D_2\Omega^2 = 0$. As the GVM increases, the resonance closer to the center frequency (Ω=0) asymptotically approaches $\Omega = \xi/D_1$ while the other resonance continues to move away from the center frequency. With ξ=-2π, k''_2=372 fs/mm^2 and L=25 mm, the behavior of the frequency-dependent nonlinear response function is divided into two distinct regimes. When the GVM is small such that $|D_1| < \sqrt{-4\xi D_2}$, there is no real root of $\xi - D_1\Omega - D_2\Omega^2 = 0$ and thus no narrowband resonance phenomenon is present near the center frequency. In this regime, the smooth profiles of $P(\Omega)$ and $Q(\Omega)$ and the relatively large bandwidth guarantee the GVM has minimal perturbative effect to the DQS. On the other hand, resonant ETPA peaks and associated nonlinear phase anomalies reappear as the GVM increase above $\sqrt{-4\xi D_2}$. Similarly, the resonance closer to the center frequency asymptotically approaches $\Omega = \xi/D_1$. The resonant ETPA peaks and associated nonlinear phase anomalies will eventually cause perturbations to soliton generation, which will be discussed in detail in Note 2.

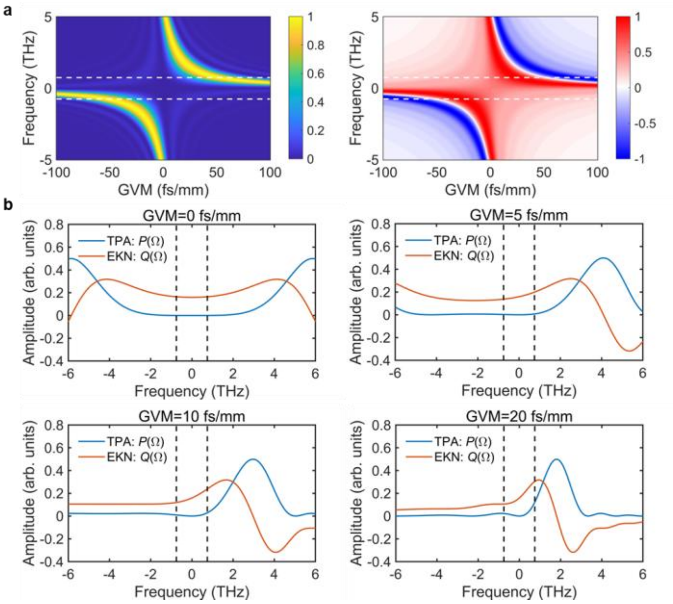

Supplementary Figure 1. Effect of GVM on the frequency response of $P(\Omega)$ and $Q(\Omega)$ with ξ=2π (a) Left: $P(\Omega)$; right: $Q(\Omega)$. (b) Line profiles of $P(\Omega)$ and $Q(\Omega)$ with different GVMs. k''_2=372 fs/mm^2 and L=25 mm. The dashed lines indicate the 3-dB soliton bandwidth of 1.5 THz, corresponding to a 200-fs sech^2 pulse.

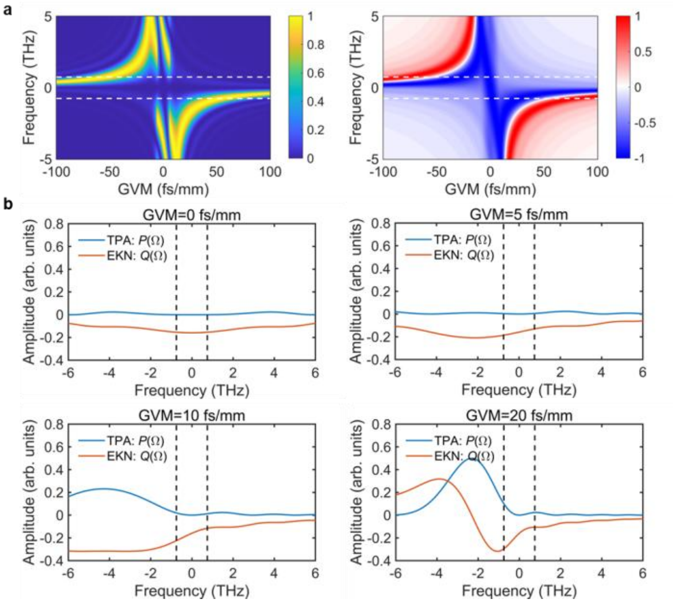

Supplementary Figure 2. Effect of GVM on the frequency response of $P(\Omega)$ and $Q(\Omega)$ with ξ=-2π (a) Left: $P(\Omega)$; right: $Q(\Omega)$. (b) Line profiles of $P(\Omega)$ and $Q(\Omega)$ with different GVMs. k''_2=372 fs/mm^2 and L=25 mm. The dashed lines indicate the 3-dB soliton bandwidth of 1.5 THz, corresponding to a 200-fs sech^2 pulse.

Similarly, in Supplementary Figs. 3 and 4 we plot the dispersive effective third-order nonlinearity as a function of the phase mismatch ξ with zero GVM. The GVM is set to be zero. When $\xi > 0$, there exists two resonant ETPA peaks and associated nonlinear phase anomalies symmetrically located at $\Omega = \pm\sqrt{\xi/D_2}$. When $\xi < 0$, both dispersive ETPA and dispersive EKN vary periodically with the phase mismatch. The effect of phase mismatch ξ on DQS will be discussed in detail in Note 3.

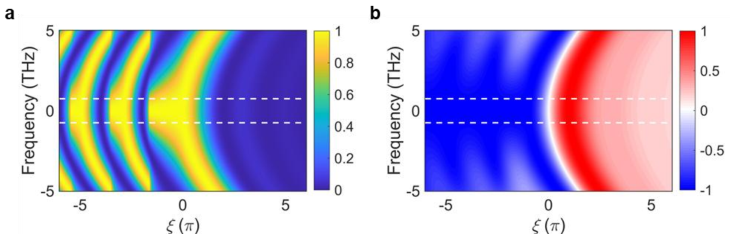

Supplementary Figure 3. Frequency response of $P(\Omega)$ (a) and $Q(\Omega)$ (b) as a function of the phase mismatch ξ. GVM=0, k''_2=372 fs/mm^2 and L=25 mm. The dashed lines indicate the 3-dB soliton bandwidth of 1.5 THz, corresponding to a 200-fs sech^2 pulse.

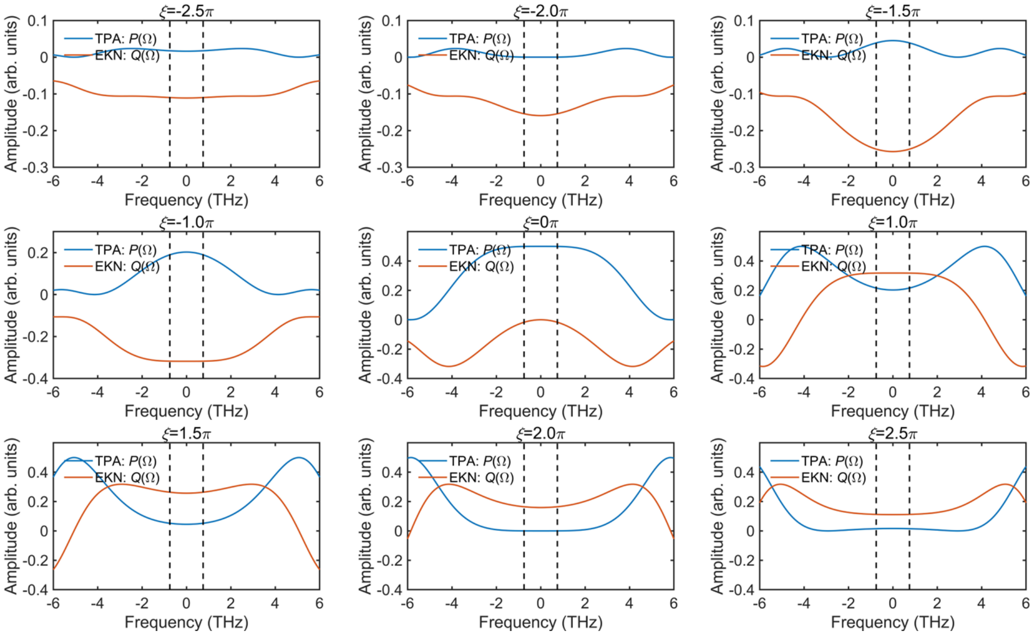

Supplementary Figure 4. Line profiles of frequency response of $P(\Omega)$ and $Q(\Omega)$ with different phase mismatch ξ. GVM=0, k''_2=372 fs/mm^2 and L=25 mm.

According to Supplementary Fig. 4, when GVM equals zero, the variations of $P(\Omega)$ and $Q(\Omega)$ over the THz bandwidth are very small, leading to near constant $P(\Omega)$ and $Q(\Omega)$. Thus, Eq. (5) can be further simplified into a similar form of conventional Lugiato-Lefever equation (LLE) by treating $P(\Omega)$ and $Q(\Omega)$ as constant values $P(0)$ and $Q(0)$ respectively:

$$t_R \frac{\partial}{\partial t} A = \left(-\alpha_1 - i\delta_1 - i\frac{k_1''L}{2}\frac{\partial^2}{\partial \tau^2}\right)A - \alpha_{ETPA} L|A|^2 A + i\gamma_{eff} L|A|^2 A + \sqrt{\theta_1} A_{in} \quad (7)$$

where $\alpha_{ETPA} = \kappa^2 LP(0) = \kappa^2 L sinc^2(\xi/2)/2$ is the ETPA coefficient and $\gamma_{eff} = \kappa^2 LQ(0) = \kappa^2 L[1 - sinc(\xi)]/\xi$ is the EKN coefficient. In the experiment, the variations of $P(\Omega)$ and $Q(\Omega)$ over the 1.5-THz bandwidth is less than 6%. With ξ=-2π, the ETPA coefficient equals zero and the EKN coefficient is more than 30 times larger than the material Kerr nonlinearity in bulk PPLN crystal, resulting in the same form with the conventional LLE.

Finally, the simulation parameters used in the main text are listed in Supplementary Table 1.

Supplementary Table 1 List of simulation parameters

Symbol	Value	Symbol	Value
L	25 mm	θ_1	0.011
α_{c1}	$0.006/L$	α_1	0.0085
α_{c2}	$0.006/L$	$\Delta k'$	0 fs/mm
$\xi=\Delta k\, L$	-2π	Δk	$-2\pi/L$
k_1''	105 fs^2/mm	k_2''	372 fs^2/mm
d_{eff}	2.7 pm/V	κ	$0.7562\ /(\sqrt{W}m)$
A_{eff}	$\pi\cdot(65\mu m)^2$	$n_{1,2}$	2.263
$\omega_0/2\pi$	190.95 THz	δ_1	0.072
c	299792458 m/s	$\Delta_1=\delta_1/\alpha_1$	-8.4706
$\lvert A_{in}\rvert^2$ or pulse peak power	20 W	ε_0	8.854E-12 F/m
Pump pulse shape	Gaussian	Pump pulse width	12 ps

2. Experimental details of the pulsed pump laser

The pulsed pump laser is a home-built electro-optic frequency comb consisting of one intensity modulator and two phase modulators driven by a 3.1-GHz RF source (Supplementary Fig. 5a). A 1/8 pulse picker is then used to reduce the repetition rate to 387 MHz that matches the DQS cavity FSR. Supplementary Fig. 5b shows the comb spectra before and after the pulse picker. The pump peak power is subsequently amplified to 20 W by an L-band EDFA, and the resulting pump spectrum is shown in Supplementary Fig. 5c, where long-wavelength ASE remains observable. Finally, Supplementary Fig. 5d shows the intensity autocorrelation of the 12-ps dechirped Gaussian pulsed pump.

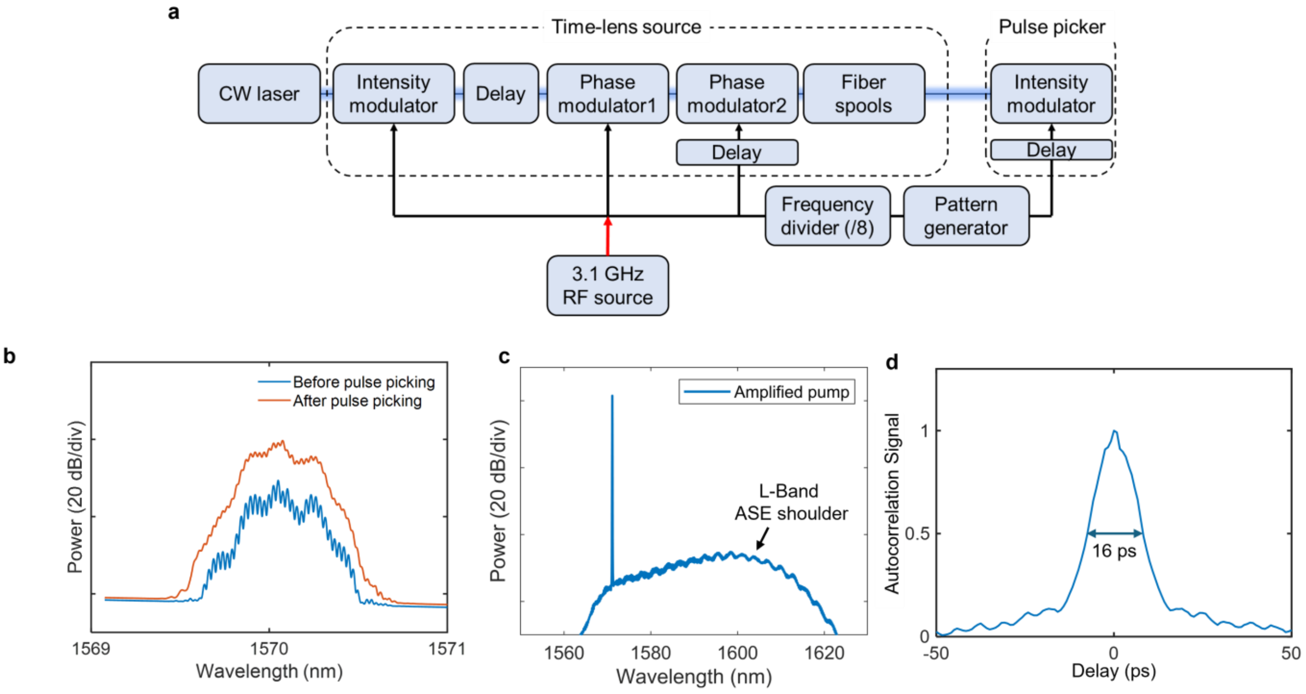

Supplementary Figure 5. (a) Schematic of the pulsed pump laser. (b) Optical spectra of the pulsed pump laser before and after the pulse picker. (c) Optical spectrum of the amplified pulsed pump. (d) Intensity autocorrelation of the 12-ps dechirped Gaussian pulsed pump.

3. Effect of group velocity mismatch on bright DQS

According to the theoretical analysis and plotted figures above, large GVM will cause detrimental narrowband perturbations to the soliton generation. A straightforward strategy is to keep the closer resonance well away from the center frequency by more than the pulse bandwidth, namely $\xi/D_1 > 0.315\pi/\Delta T$ (assuming a sech^2 pulse shape), through the choice of large phase mismatch ξ at the cost of reduced effective Kerr nonlinearity.

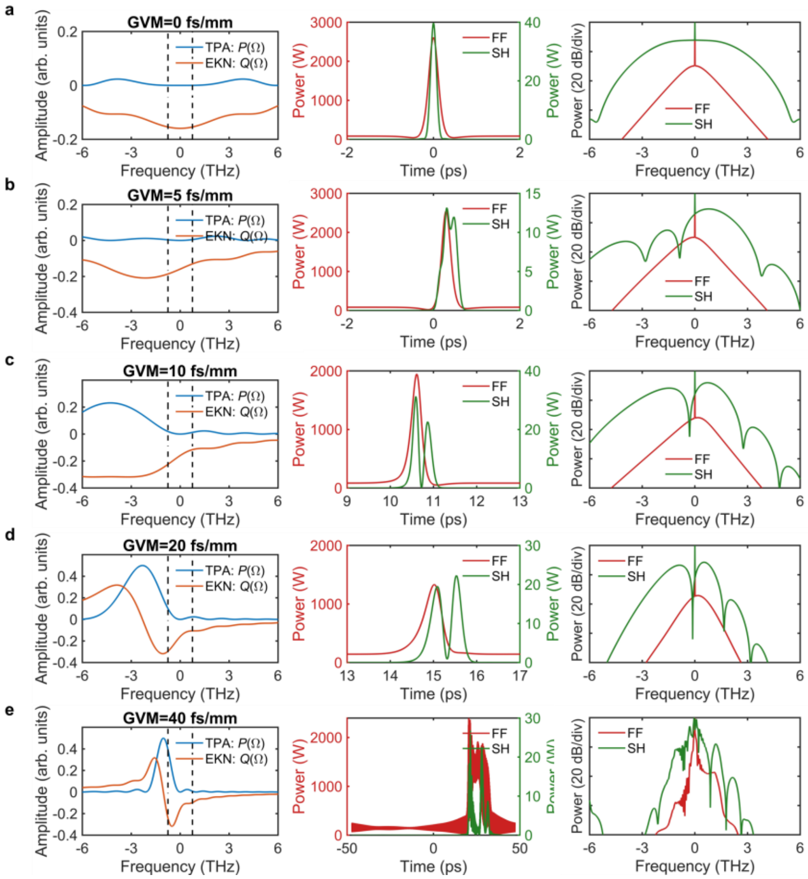

Supplementary Figure 6. Effect of GVM on frequency response of $P(\Omega)$ and $Q(\Omega)$ (left), pulse profile (middle) and spectrum (right) with phase mismatchs ξ=-2π, k''_2=372 fs/mm^2 and L=25 mm. (a) GVM = 0 fs/mm; (b) GVM = 5 fs/mm; (c) GVM = 10 fs/mm; (d) GVM = 20 fs/mm; (e) GVM = 40 fs/mm.

Supplementary Figure 6 plots the effect of GVM on DQS pulse profile and spectrum under CW pump utilizing coupled two-wave equation. With large GVM, asymmetry is evidently observed in both the FF pulse profile and the corresponding optical spectrum, similar to the Raman effect caused DKS pulse distortion and center frequency shift [4]. Moreover, large temporal walk-off between the SH field and FF lead to SH pulse splitting and apparent SH spectral fringes similar to the soliton fission dynamics [5]. When the GVM $\Delta k'$ approaches 40 fs/mm, the perturbation grows so strong that the EKN and especially the ETPA coefficients change intensively and cannot be viewed as constant within the soliton bandwidth. Therefore, there exhibits no soliton generation but only unstable frequency combs arising from walk-off induced modulation instability.

In the experiment, the zero-GVM wavelength is set to be ~1571 nm at a PPLN crystal temperature of 28.7°C (Supplementary Fig. 7). Moreover, we adjust the FF wavelength to efficiently tune the GVM between SH field and FF with a coefficient of 0.5 fs/mm/nm. Besides, we can also change the GVM by tuning the crystal temperature with a coefficient of 0.62 fs/mm/°C. However, since the phase mismatch strongly depends on the crystal temperature, the poling period of the PPLN crystal might be changed to fulfill the original phase matching condition.

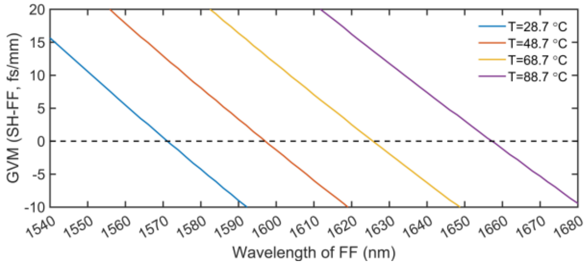

Supplementary Figure 7. GVM dependence on the FF wavelength at different PPLN crystal temperatures.

4. Effect of phase mismatch on bright DQS

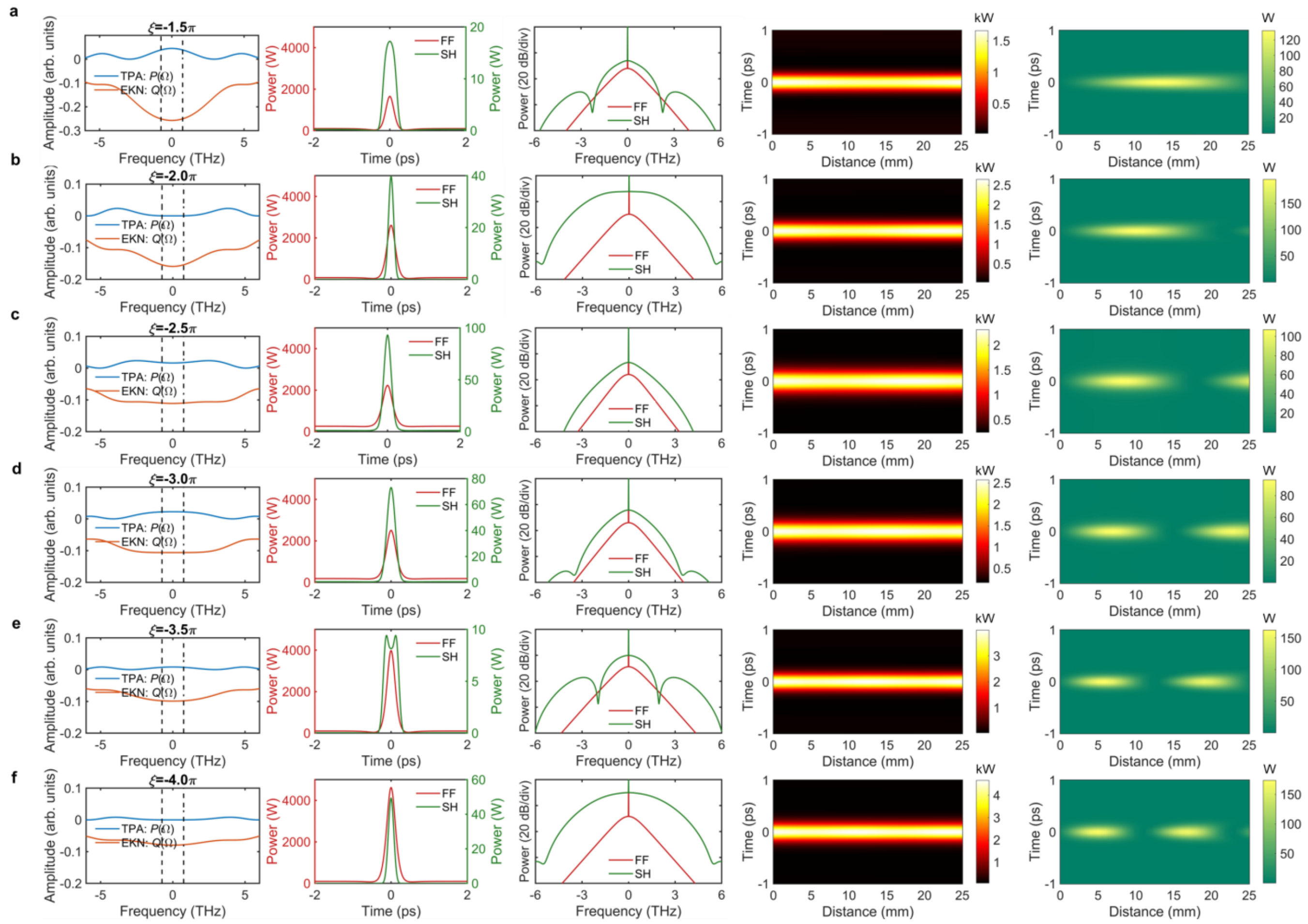

Supplementary Figure 8. Effect of phase mismatchs ξ on bright DQS generation. The first column is the frequency response of $P(\Omega)$ and $Q(\Omega)$ (left), the second column is the pulse profile at the output facet of the PPLN crystal, the third column is the spectrum at the output facet of the PPLN crystal, the fourth column is the FF pulse evolution inside the PPLN crystal, the fifth column is the SH pulse evolution inside the PPLN crystal. GVM = 0, k''_2=372 fs/mm^2 and L=25 mm. (a) ξ=-1.5π; (b) ξ=-2.0π; (c) ξ=-2.5π; (d) ξ=-3.0π; (e) ξ=-3.5π; (f) ξ=-4.0π.

Supplementary Figures 8 plots the effect of phase mismatch on DQS under CW pump utilizing coupled two-wave equation. In theory, the phase mismatchs ξ determines back conversion between the two fields, including the conversion strength and conversion speed. According to pulse evolution inside the PPLN crystal, to fulfill the self-reproduction condition the FF at both input and output facet remain the same with sech^2 profiles but different bandwidths due to the different conversion strength. However, the unresonant SH field always starts from vacuum noise at the input facet of the PPLN crystal for different phase mismatchs ξ, while it varies a lot at the output facet due to the different conversion speeds. The SH pulse profiles vary from single smooth pulse to saddle-splitting pulse, while the SH spectra vary from flat-top profile, sech^2 profile, parabolic profile to profiles with many fringes. Of note, for all cases, the dispersive ETPA and EKN coefficients can be viewed as constant especially within the soliton bandwidth (3 dB). Therefore, the dispersion of ETPA and EKN coefficients cannot explain different SH fields at the output facet.

In the experiment, we adjust the PPLN crystal temperature to efficiently tune the phase mismatch ξ with a coefficient of ~10π/°C for the 25-mm-long PPLN bulk crystal (Supplementary Fig. 9). Moreover, the phase mismatch ξ varies little for different wavelengths from 1550 nm to 1580 nm, also verified the near-zero GVM around 1570 nm.

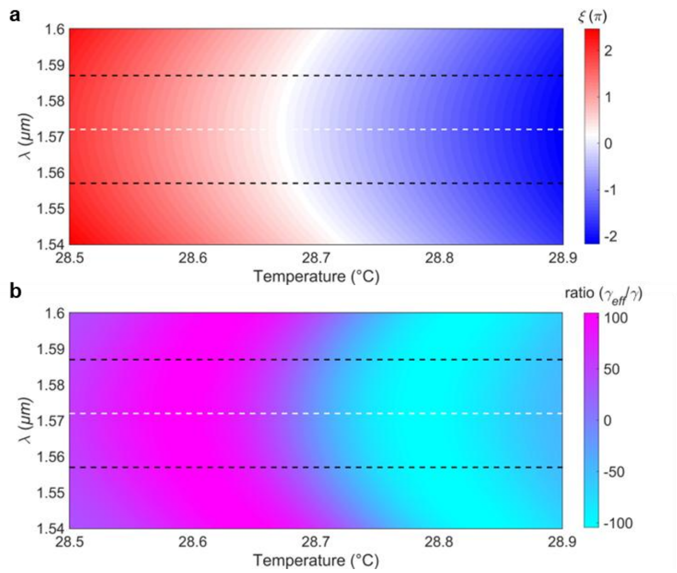

Supplementary Figure 9. PPLN crystal temperature influences the phase mismatch ξ (a) thus the effective Kerr nonlinearity (b). The white dashed line indicates the zero-GVM wavelength at ~1571 nm. Crystal length is L=25 mm.

5. Effect of group velocity dispersion on bright DQS

Supplementary Figures 10 plots the effect of SH GVD k''_2 on DQS under CW pump utilizing coupled two-wave equation. Compared to the GVM, the SH GVD induces a second-order perturbation to $x(\Omega) = \xi - D_1\Omega - D_2\Omega^2$. With GVM=0, $x(\Omega) = \xi - D_2\Omega^2$. When $\xi D_2 < 0$, there are two resonant effective TPA peaks and associated nonlinear phase anomalies close to the center frequency, which cause SH pulse splitting and spectral fringes. When $\xi D_2 > 0$, the smooth profiles of $P(\Omega)$ and $Q(\Omega)$ and the relatively large bandwidth guarantee the SH GVD has minimal perturbative effect to the quadratic soliton.

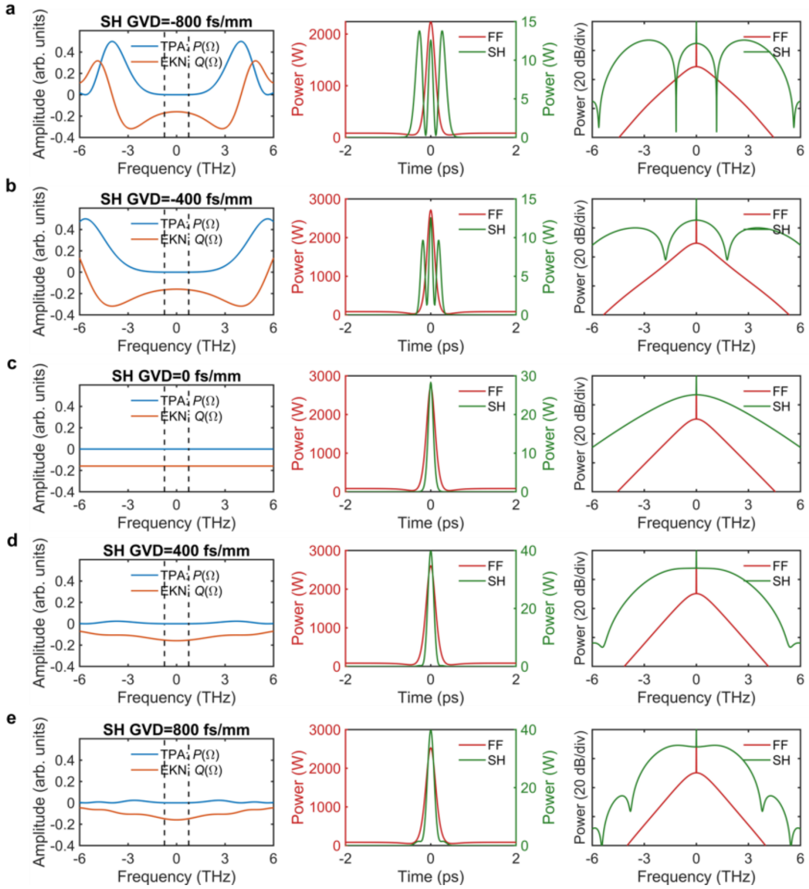

Supplementary Figure 10. Effect of SH GVD k''_2 on frequency response of $P(\Omega)$ and $Q(\Omega)$ (left), pulse profile (middle) and spectrum (right) with phase mismatchs ξ=-2π, GVM=0 and L=25 mm. (a) k''_2 = -800 fs^2/mm; (b) k''_2 = -400 fs^2/mm; (c) k''_2 = 0 fs^2/mm; (d) k''_2 = 400 fs^2/mm; (e) k''_2 = 800 fs^2/mm.

In terms of FF GVD, we study its effect on both the FF and SH field as shown in Supplementary Fig. 11. In general, the FF GVD affects the DQS bandwidth. Small FF GVD leads to larger FF bandwidth as in the conventional DKS case. Moreover, small FF GVD also leads to larger SH spectral bandwidth, due to the mutual trapping between the two pulses.

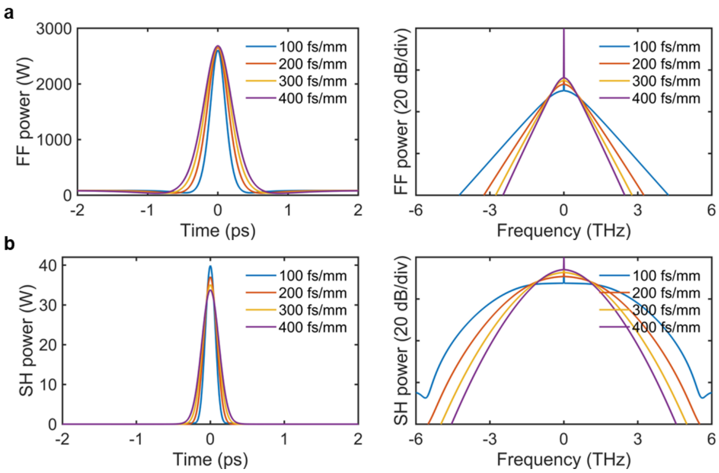

Supplementary Figure 11. Effect of FF GVD on DQS performance. (a) FF; (b) SH field. Left: temporal profile; right: spectral profile. ξ=-2π， GVM=0, k''_2=372 fs/mm^2 and L=25 mm.

6. Single sideband phase noise spectra of DQS in different states

The single-sideband (SSB) phase noise spectra of DQS in different states are plotted in Supplementary Fig. 12. Due to the mutual trapping between FF and SH DQS, a good overlap between their SSB phase noise spectra was observed. For offset frequencies below 70 kHz, the bright DQS exhibits higher phase noise than the platicon, whereas at higher offset frequencies, its phase noise becomes lower. Furthermore, the phase noise of both the bright DQS and the platicon is higher than that of the pump. One possible explanation is that the excessive phase noise arises from cavity-length fluctuations, as the pump repetition rate was not actively stabilized to the cavity FSR in our setup. Another possible explanation is that the excess phase noise is induced by the injected ASE of the L-band EDFA (Supplementary Fig. 5c). A dedicated and comprehensive follow-up study is required to elucidate the physical origin of the observed phase noise behavior.

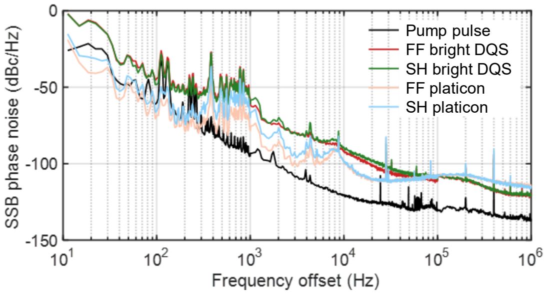

Supplementary Figure 12. SSB phase noise spectra of DQS in different states.

7. Intensity autocorrelation of bright DQS and platicon

Supplementary Figure 13a shows the optical spectrum of the single bright DQS after removing the pump background with a volume Bragg grating (VBG), and Supplementary Fig. 13b presents the corresponding intensity autocorrelation trace, confirming its femtosecond pulse characteristics. Supplementary Figure 13c shows the optical spectrum of the platicon in state I at a normalized detuning of 5.7, and Supplementary Fig. 13d presents the corresponding intensity autocorrelation trace, confirming its flat-top temporal profile.

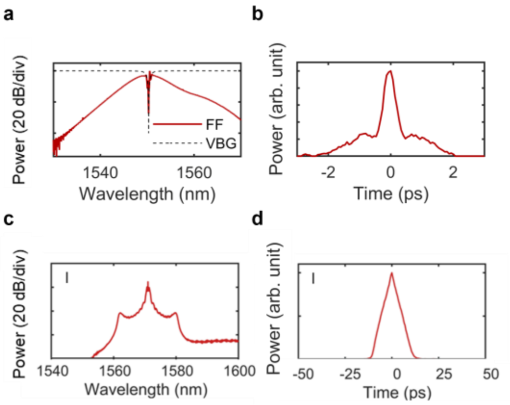

Supplementary Figure 13. (a) Optical spectrum of the single bright DQS with the pump background removed by VBG. (b) The corresponding intensity autocorrelation trace. (c) Optical spectrum of the platicon in state I with a normalized detuning of 5.7. (d) The corresponding intensity autocorrelation trace.

8. Green frequency comb generated through SFG between the FF and SH

Due to the high peak power of the generated platicon, green frequency combs resulting from SFG of the FF and SH in the PPLN crystal (1571 nm + 785.5 nm →524 nm) are also observed as shown in Supplementary Fig. 14.

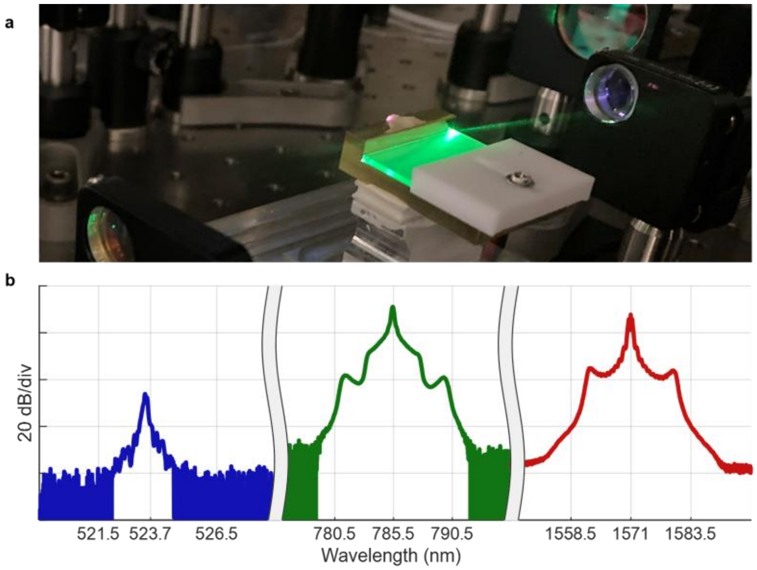

Supplementary Figure 14. (a) Picture of green laser emitted from PPLN crystal; (b) Optical spectrum of generated green laser (left), platicon of the SH field (middle) and platicon of the FF (right).

9. Simulated DQS generation dynamics with a pulsed pump

Supplementary Figure 15 shows the simulated bright DQS generation dynamics under a 12-ps pulsed pump when the FF frequency is scanned from the red to blue detuning. In particular, when a CW component coexists with the pulsed pump owing to the limited extinction ratio of pulse picking, a pronounced peak appears in the FF intracavity average power near zero detuning, consistent with the experimental observation (main Fig. 3a).

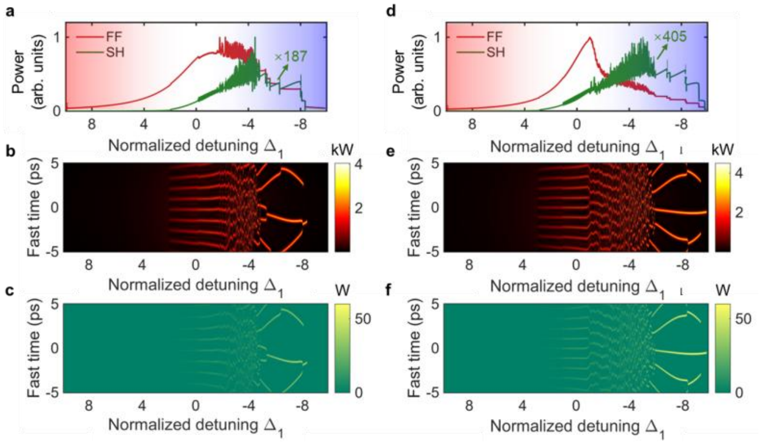

Supplementary Figure 15. (a) Intracavity average power of the FF and SH, (b) temporal evolution of the FF, and (c) temporal evolution of the SH as a function of normalized detuning under a 12-ps pulsed pump. The SH power is scaled by 187 for clarity. (d) Intracavity average power of the FF and SH, (e) temporal evolution of the FF, and (f) temporal evolution of the SH as a function of normalized detuning under a 12-ps pulsed pump with a coexisting CW component. The SH power is scaled by 405 for clarity.

Supplementary Figure 16 shows the simulated bright DQS generation dynamics under a 12-ps pulsed pump when the FF frequency is scanned from the blue to red detuning. In particular, when a CW component coexists with the pulsed pump owing to the limited extinction ratio of pulse picking, a pronounced peak appears in the FF intracavity average power near zero detuning, consistent with the experimental observation (Supplementary Fig. 16g).

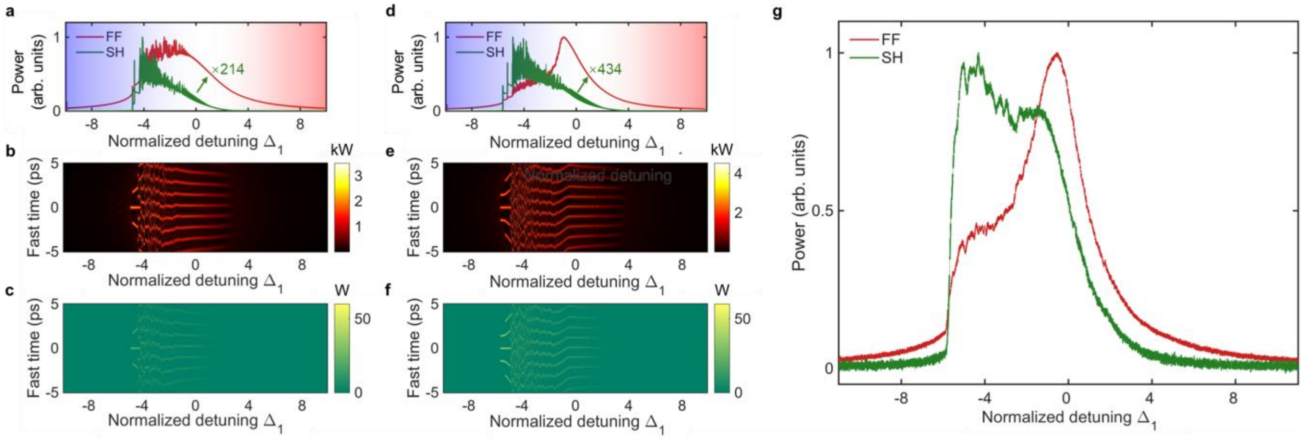

Supplementary Figure 16. (a) Intracavity average power of the FF and SH, (b) temporal evolution of the FF, and (c) temporal evolution of the SH as a function of normalized detuning under a 12-ps pulsed pump. The SH power is scaled by 214 for clarity. (d) Intracavity average power of the FF and SH, (e) temporal evolution of the FF, and (f) temporal evolution of the SH as a function of normalized detuning under a 12-ps pulsed pump with a coexisting CW component. The SH power is scaled by 434 for clarity. (g) Experimentally measured output average power of the FF and SH as a function of normalized detuning.

Supplementary Figure 17 shows the simulated platicon generation dynamics under a 12-ps pulsed pump when the FF frequency is scanned from the red to blue detuning. In particular, when a CW component coexists with the pulsed pump owing to the limited extinction ratio of pulse picking, a pronounced peak appears in the FF intracavity average power near zero detuning, consistent with the experimental observation (main Fig. 4a).

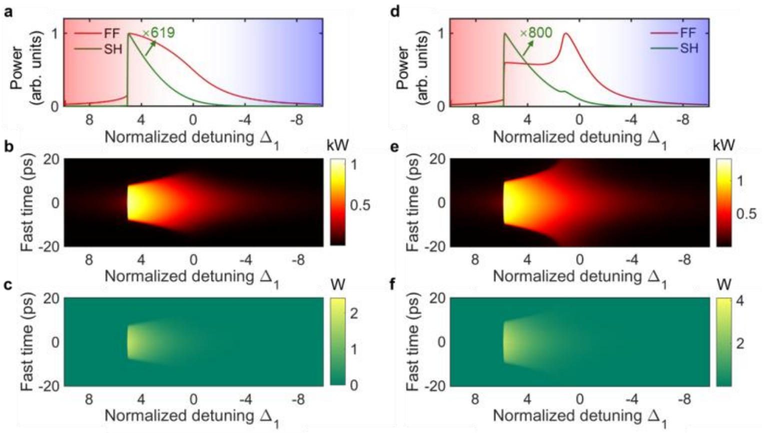

Supplementary Figure 17. (a) Intracavity average power of the FF and SH, (b) temporal evolution of the FF, and (c) temporal evolution of the SH as a function of normalized detuning under a 12-ps pulsed pump. The SH power is scaled by 619 for clarity. (d) Intracavity average power of the FF and SH, (e) temporal evolution of the FF, and (f) temporal evolution of the SH as a function of normalized detuning under a 12-ps pulsed pump with a coexisting CW component. The SH power is scaled by 800 for clarity.

Supplementary Figure 18 shows the simulated platicon generation dynamics under a 12-ps pulsed pump when the FF frequency is scanned from the blue to red detuning. In particular, when a CW component coexists with the pulsed pump owing to the limited extinction ratio of pulse picking, a pronounced peak appears in the FF intracavity average power near zero detuning, consistent with the experimental observation (Supplementary Fig. 18g).

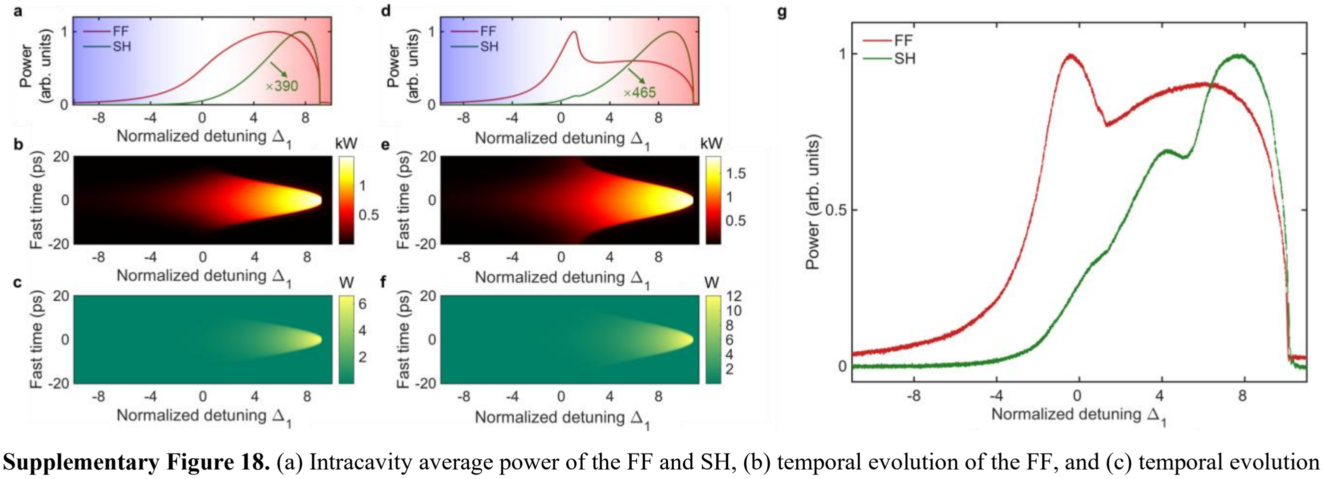

Supplementary Figure 18. (a) Intracavity average power of the FF and SH, (b) temporal evolution of the FF, and (c) temporal evolution of the SH as a function of normalized detuning under a 12-ps pulsed pump. The SH power is scaled by 390 for clarity. (d) Intracavity average power of the FF and SH, (e) temporal evolution of the FF, and (f) temporal evolution of the SH as a function of normalized detuning under a 12-ps pulsed pump with a coexisting CW component. The SH power is scaled by 465 for clarity. (g) Experimentally measured output average power of the FF and SH as a function of normalized detuning.

10. Effect of group velocity mismatch and phase mismatch on platicon with a pulsed pump

Supplementary Figure 19 shows that the GVM causes asymmetry on both the DQS pulse shape and DQS spectrum especially for the SH soliton mainly due to the asymmetric dispersive EKN and ETPA coefficients. The phase mismatch leads to the FF soliton bandwidth change and SH soliton variation (Supplementary Fig. 20) mainly due to the change of strength and speed in the back conversion, similar to the discussion regarding Supplementary Fig. 8.

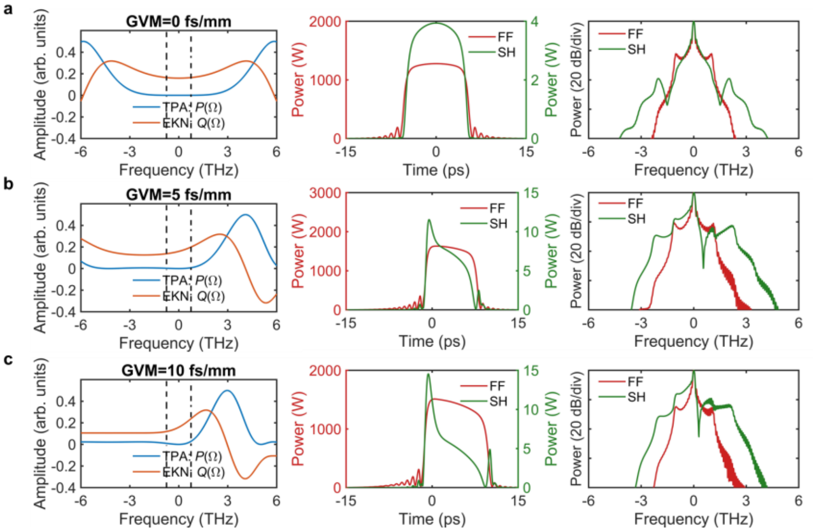

Supplementary Figure 19. Effect of GVM on frequency response of $P(\Omega)$ and $Q(\Omega)$ (left), pulse profile (middle) and spectrum (right) with phase mismatchs $\xi=2\pi$, $k''_2=372$ fs/mm^2 and L=25 mm. (a) GVM = 0 fs/mm; (b) GVM = 5 fs/mm; (c) GVM = 10 fs/mm.

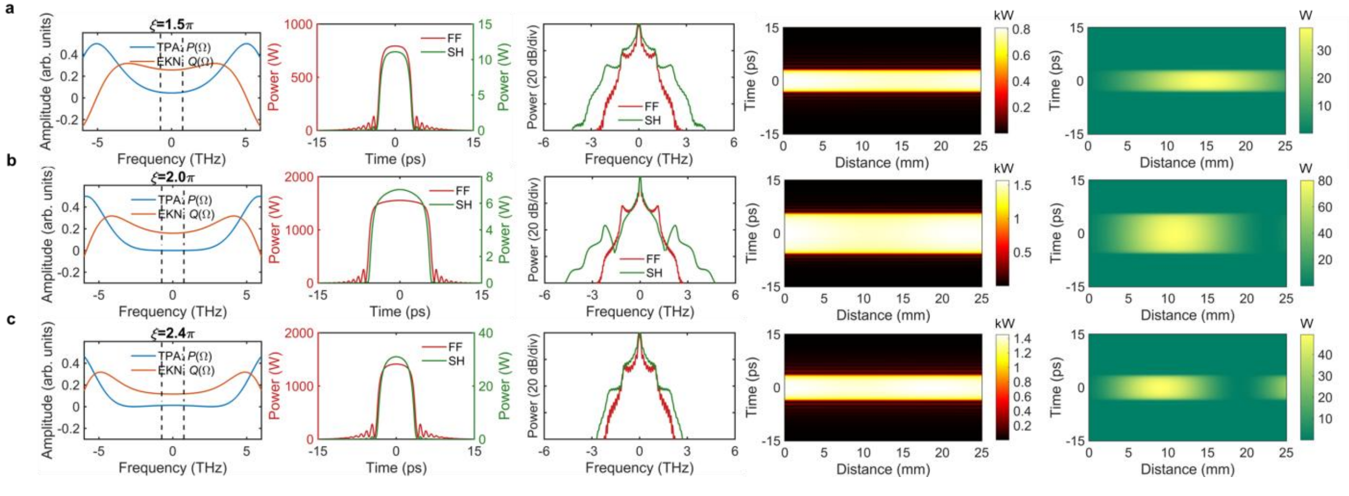

Supplementary Figure 20. Effect of phase mismatch ξ on platicon generation. The first column is the frequency response of $P(\Omega)$ and $Q(\Omega)$ (left), the second column is the pulse profile at the output facet of the PPLN crystal, the third column is the spectrum at the output facet of the PPLN crystal, the fourth column is the FF pulse evolution inside the PPLN crystal, the fifth column is the SH pulse evolution inside the PPLN crystal. GVM = 0, $k''_2=372$ fs/mm^2 and L=25 mm. (a) $\xi=1.5\pi$; (b) $\xi=2.0\pi$; (c) $\xi=2.4\pi$.

11. Effect of pump desynchronization on bright DQS and platicon

The bright DQS exhibits strong sensitivity to pump desynchronization. A mismatch as small as 200 Hz between the pump repetition rate and the 387 MHz cavity FSR is sufficient to destabilize bright DQS formation. In contrast, the platicon remains stable under pump desynchronization up to 2 kHz. The effect of desynchronization manifests as asymmetry in the platicon optical spectrum, as shown in Supplementary Fig. 21 [6].

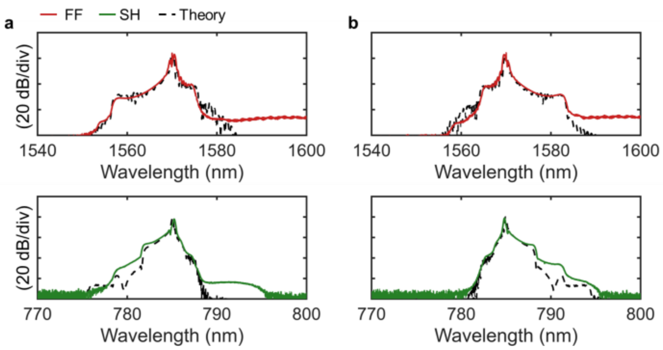

Supplementary Figure 21. (a) Optical spectra of the platicon at the FF and SH under a pump desynchronization of 2 kHz. (b) Optical spectra of the platicon at the FF and SH under a pump desynchronization of -2 kHz.

12. Effect of pump pulse shape on bright DQS and platicon

Supplementary Fig. 22 shows the effect of pump pulse shape on the bright DQS and platicon. While the pump shape has minimal impact on the spectral and temporal characteristics of the bright DQS, its effect on the platicon is more pronounced, reflecting their distinct formation mechanisms.

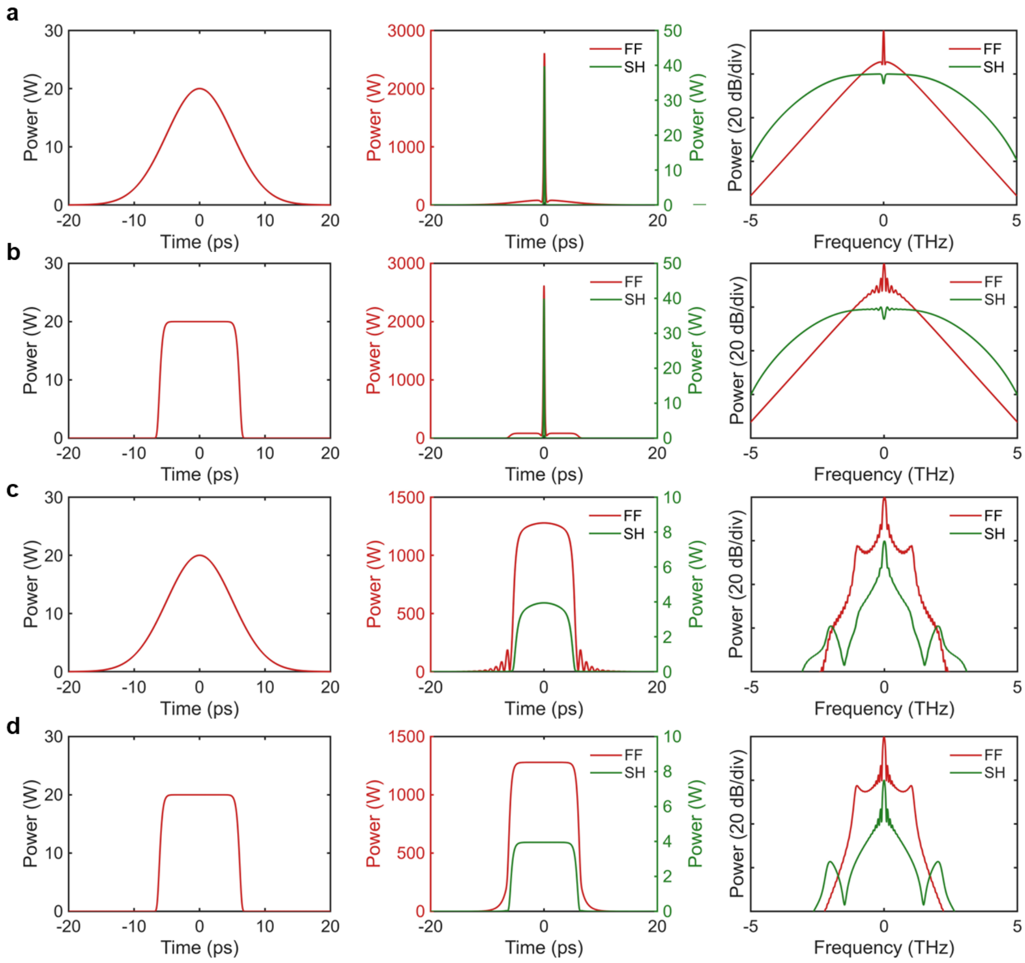

Supplementary Figure 22. (a) Bright DQS generation with a Gaussian pulse. (b) Bright DQS generation with a flat-top pulse. (c) Platicon generation with a Gaussian pulse. (d) Platicon generation with a flat-top pulse. The first column shows the temporal profiles of the pump pulses, the second column shows the temporal profiles of the generated pulses, and the third column shows their corresponding optical spectra. The pump pulse duration is fixed at 12 ps for all cases.

13. Trapping of bright DQS by pulsed pump

To investigate the DQS trapping dynamics, we numerically solve the CTWE (Eqs. 1-4) using a perturbed initial condition approximating a DQS that is offset from the pulsed pump peak. This perturbation evolves into a DQS that may drift under the influence of the pump's amplitude gradient. The simulation continued until a steady state was reached. Supplementary Fig. 23 summarizes the results. The DQSs are generally attracted toward the edge of the pulsed pump, with their equilibrium positions dependent on the pump power. This trapping behavior closely resembles that observed in conventional pulse-driven DKSs (Supplementary Fig. 24) [7].

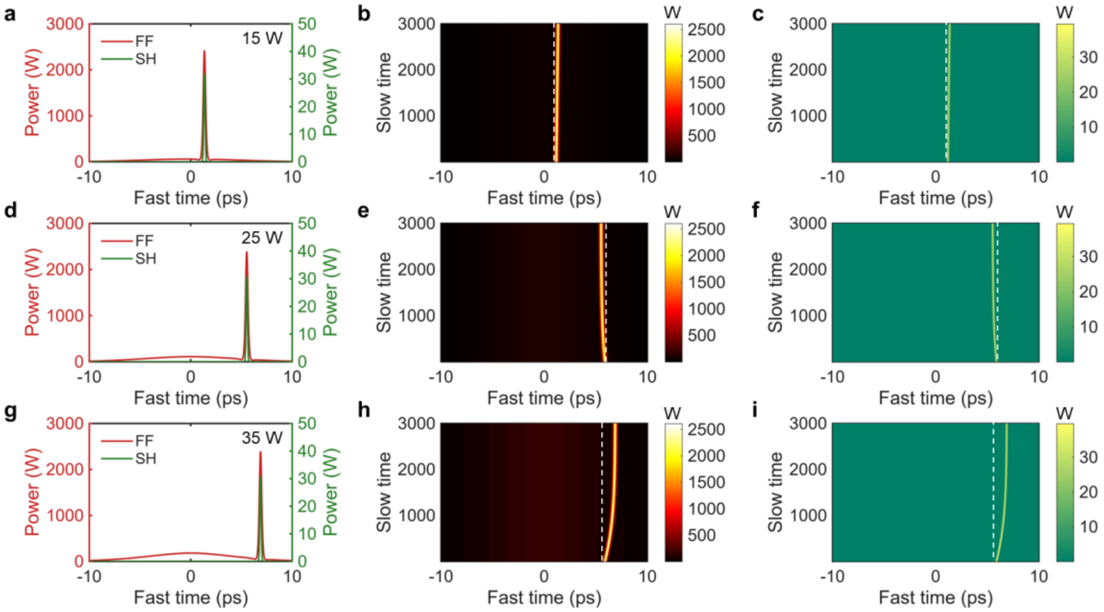

Supplementary Figure 23. (a) Steady-state DQS temporal profiles, (b) temporal evolution of the FF, and (c) temporal evolution of the SH, obtained for a pump peak power of 15 W. (d) Steady-state DQS temporal profiles, (e) temporal evolution of the FF, and (f) temporal evolution of the SH, obtained for a pump peak power of 25 W. (g) Steady-state DQS temporal profiles, (h) temporal evolution of the FF, and (i) temporal evolution of the SH, obtained for a pump peak power of 35 W.

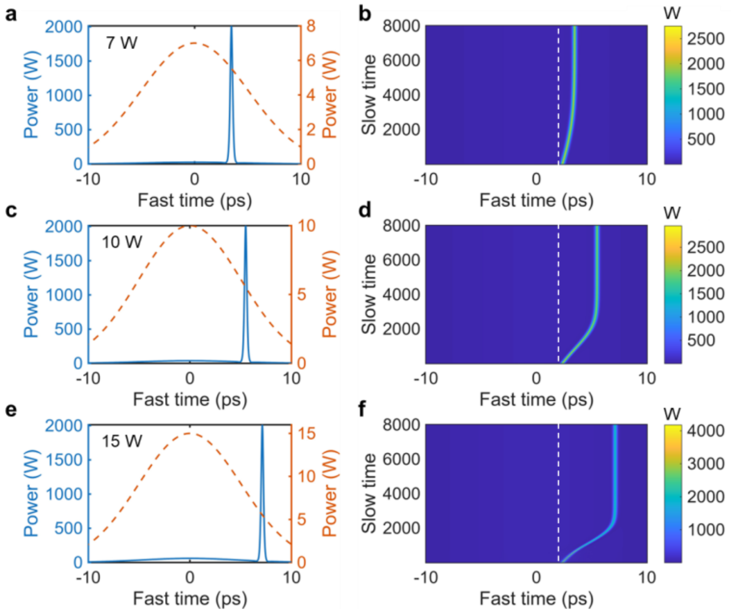

Supplementary Figure 24. (a) Steady-state DKS temporal profiles, and (b) temporal evolution of the DKS, obtained for a pump peak power of 7 W. (c) Steady-state DKS temporal profiles, and (d) temporal evolution of the DKS, obtained for a pump peak power of 10 W. (e) Steady-state DKS temporal profiles, and (f) temporal evolution of the DKS, obtained for a pump peak power of 15 W.

Supplementary References

[1] F. Leo, T. Hansson, I. Ricciardi, M. De Rosa, S. Coen, S. Wabnitz, M. Erkintalo, Walk-off-induced modulation instability, temporal pattern formation, and frequency comb generation in cavity-enhanced second-harmonic generation, Phys. Rev. Lett. 116 (2016) 033901.

[2] M. Nie, S.-W. Huang, Quadratic solitons in singly resonant degenerate optical parametric oscillators, Phys. Rev. Appl. 13 (2020) 044046.

[3] M. Nie, Y. Xie, B. Li, S.-W. Huang, Photonic frequency microcombs based on dissipative Kerr and quadratic cavity solitons, Prog. Quantum Electron. (2022) 100437. https://doi.org/10.1016/j.pquantelec.2022.100437.

[4] M. Karpov, H. Guo, A. Kordts, V. Brasch, M.H. Pfeiffer, M. Zervas, M. Geiselmann, T.J. Kippenberg, Raman self-frequency shift of dissipative Kerr solitons in an optical microresonator, Phys. Rev. Lett. 116 (2016) 103902.

[5] L. Yin, Q. Lin, G.P. Agrawal, Soliton fission and supercontinuum generation in silicon waveguides, Opt. Lett. 32 (2007) 391–393.

[6] M. Macnaughtan, M. Erkintalo, S. Coen, S. Murdoch, Y. Xu, Temporal characteristics of stationary switching waves in a normal dispersion pulsed-pump fiber cavity, Opt. Lett. 48 (2023) 4097–4100.

[7] I. Hendry, W. Chen, Y. Wang, B. Garbin, J. Javaloyes, G.-L. Oppo, S. Coen, S.G. Murdoch, M. Erkintalo, Spontaneous symmetry breaking and trapping of temporal Kerr cavity solitons by pulsed or amplitude-modulated driving fields, Phys. Rev. A 97 (2018) 053834.
